# Opportunities to engage health system leaders in whole systems approaches to physical activity in England

**DOI:** 10.1186/s12889-022-12602-5

**Published:** 2022-02-08

**Authors:** E. L. Bird, D. Evans, S. Gray, E. Atherton, J. Blackshaw, M. Brannan, N. Corrigan, D. Weiner

**Affiliations:** 1 Centre for Public Health and Wellbeing, University of the West of England, Frenchay Campus, Bristol, BS16 1QY UK; 2 Office for Health Improvement and Disparities, Department of Health and Social Care, 39 Victoria Street, London, SW1H 0EU UK; 3 Office for Health Improvement and Disparities, Department of Health and Social Care, Blenheim House, West One, Duncombe Street, Leeds, LS1 4PL UK

**Keywords:** Whole systems approaches, Physical activity, Systems leadership, Public health, Healthcare public health

## Abstract

**Background:**

Physical activity plays an important role in maintaining good health and wellbeing, non-communicable disease prevention and can improve healthcare outcomes. Some progress is being made on incorporating physical activity into routine care, but less on engaging health system leaders in the ‘whole systems’ approaches which are increasingly recognised as important for addressing complex public health challenges such as physical inactivity. This commentary builds upon the findings of a recent study and aims to identify opportunities for engaging National Health Service (NHS) systems leaders in whole systems approaches to physical activity.

**Opportunities for action in England:**

Pockets of good practice exist from which lessons can be learned, but there are systemic issues that discourage and create barriers, and a need for meaningful engagement, leadership and action at national, regional and local levels. National and regional actors like Sport England, NHS England, health professional bodies, Active Partnerships, the Local Government Association and the Office for Health Improvement and Disparities can encourage and support government and the NHS to change policy drivers, culture and practices. Emerging opportunities include the 2021 White Paper *Integration and Innovation*, development of local integrated care systems, leadership from health charities and investment in non-clinical interventions (‘social prescribing’). At local level, public health and physical activity specialists and other organisations have a key role as champions and facilitators of local whole systems approaches and engagement of local NHS leaderships. Finally, although whole systems action is about collaborative leadership, individual champions of physical activity can make a difference in influencing NHS leaders at every level towards whole systems working.

**Supplementary Information:**

The online version contains supplementary material available at 10.1186/s12889-022-12602-5.

## Background

Persistently high levels of physical inactivity constitute a public health problem globally [1]. Physical inactivity contributes to chronic diseases which are a burden to health care systems such as the National Health Service (NHS) in England [2, 3]. There remain stark inequalities when it comes to how physically active people are and the following groups are particularly affected - older people, people living in more deprived areas, people living with long term conditions and/or physical or learning disabilities and, people in Black, Asian and Minority Ethnic groups [4]. More positively, increasing physical activity has been shown to improve health and wellbeing at every age and in diverse populations [5, 6].

Growing recognition of the issue of physical inactivity has resulted in the publication of numerous policy drivers and strategies advocating for physical activity, including the World Health Organization’s *Global Action Plan on Physical Activity 2018–2030 - More Active People for a Healthier World* [7]. Investment in policies that promote physical activity can contribute positively to achieving many of the 2030 United Nations Sustainable Development Goals [7, 8]. In 2013, the World Health Organization pledged to reduce global physical inactivity prevalence by 10% by 2025 [9]. However, findings from a pooled analysis of population based survey data suggest that if current trends continue the target will not be met [10].

The COVID-19 pandemic and the necessary measures to suppress the virus has resulted in further reductions in population physical activity levels, particularly for communities with the highest health needs [11, 12]. It has led to calls for an update to the Global Action Plan on Physical Activity (GAPPA) to reflect the ‘new’ state of physical activity [13]. Arguably, COVID-19 has made physical activity promotion even more urgent and provides significant opportunities for renewed action in this area.

Tackling low levels of physical activity has been an identified priority of successive UK governments (responsible for health policy in England), resulting in the publication of numerous documents, including as a legacy commitment of the London 2012 Games, [14, 15] and the UK government’s Advancing Our Health: prevention in the 2020s Paper [16]. Some progress has been made in tackling low levels of physical activity, though it has been slow and inequalities remain for people with health issues and other groups [17]. Notably, a recent critical review of national physical activity policies in England identified 54 policy documents in circulation relating to children and young people alone [18]. This suggests that there remains a need to better connect and align policies and strategies and how best to coherently disseminate and communicate key messages to various audiences. In 2021 Sport England, a UK government body responsible for growing and developing grassroots sport and getting more people active across England published its timely *Uniting the Movement* strategy [19]. The strategy prioritises connecting physical activity with health and wellbeing as critical to get the population active, and provides a real moment to harness and align action through a long-term whole systems approach. There is also increasing political recognition of the importance of the link between physical activity and population health and wellbeing, including a call for a ‘national sport, health and wellbeing plan’ and creation of a new ministerial post with accountability within the Department of Health and Social Care [20].

### Whole systems approaches to physical activity

There is growing interest in the public health field in whole systems approaches to population health in general, [21] and such approaches related to promoting physical activity in particular [22, 23]. Rutter and colleagues in 2017 described a complex systems model of public health in which poor health and health inequalities are conceptualised “as outcomes of a multitude of interdependent elements within a connected whole. These elements affect each other in sometimes subtle ways, with changes potentially reverberating throughout the system” [24]. This was followed in 2019 by a paper calling for whole systems approaches to global and national physical activity plans [22].

The Global Action Plan on Physical Activity suggests that whole systems approaches are needed to combine upstream policy actions, which aim to improve social, cultural, economic and environmental factors that support physical activity, with downstream individually-focused approaches [7] It also highlights the need to scale-up policy actions and government strategies for physical activity. Whole systems approaches are arguably relevant and necessary to address low levels of physical activity, associated negative health impacts and wider outcomes in at-risk population groups including people from more disadvantaged communities. Physical inactivity intersects with a range of inequality issues associated with environments that discourage physical activity, including lack of access to green spaces, transport planning traditionally focused on car travel, lack of adequate infrastructure for walking and cycling and cultural attitudes towards physical activity [25].

In England, the Office for Health Improvement and Disparities (OHID), has responsibility for promoting physical activity and health (and has a lead role in promoting whole systems approaches to physical activity through the *Everybody Active, Every Day* framework [26]). Prior to its cessation, Public Health England had invested in systems thinking and in 2019 published *Whole systems approach to obesity: a guide to support local approaches to promoting a healthy weight* that, though focused on obesity, included the promotion of physical activity within its scope [27]. The development of the *Everybody Active, Every Day* framework [26] involved over 1000 national and local stakeholders to help address inactivity and increase physical activity. The framework promotes a whole systems cross-sector approach across four domains – active society; moving professionals; active environments; and, moving at scale. Shortly afterward the UK government published its strategy for sport and physical activity, *Sporting Future*, which for the first time identified improvement of health and achievement of the UK Chief Medical Officer’s guidelines on physical activity as specific ambitions [28].

In recent years, at a national level in England there has been a growing collaboration across sectors led by OHID and Sport England, which has tested opportunities to enable the NHS and healthcare professionals to do more to promote physical activity as part of routine care [29]. For example, Sport England have recently been working on local delivery pilots (LDPs), where 12 LDPs across England have developed and piloted whole system approaches to physical activity in local communities [30]. At a more regional level, it is pertinent to note the work in Sheffield and the Move More Sheffield whole systems approach which has helped inform ‘what works’ to promote physical activity through the co-location of healthcare and leisure settings [31, 32].

There exist many different perspectives on whole-systems approaches, with systems language sometimes used differently. This definition describes how whole systems approaches are understood in this paper:

‘A local whole systems approach responds to complexity through an ongoing, dynamic and flexible way of working. It enables local stakeholders, including communities, to come together, share an understanding of the reality of the challenge, consider how the local system is operating and where there are the greatest opportunities for change. Stakeholders agree actions and decide as a network how to work together in an integrated way to bring about sustainable, long-term systems change.’ [33]

### The NHS, whole systems approaches and systems leadership

In England there is a concern for the health of the nation and the impact that an ageing and increasingly unhealthy population has on the financial sustainability of the NHS and wider public sector [34]. A recent LSE-Lancet Commission on the future of the NHS highlighted the unsustainability of the service and the critical need to focus on improving the population’s health through prevention and health promotion [35]. This echoes the previous *NHS Long Term Plan*, [36] which called for a step change in action on prevention and a shift of resources from acute care to prevention. There remain challenges when it comes to public health funding and so whilst the latest public health budgets for 2021–22 in England represent a numeric increase, [37] budgets remain substantially lower than in 2015 [38–40].

Historically service delivery metrics such as performance, quality and finances have been prioritised as key performance indicators rather than prevention of ill-health and promotion of health and wellbeing [41]. Research indicates that supply of healthcare and, presumably to some degree, the actions of NHS system leaders are driven by responding to government decision and policy choices and, perhaps to a lesser degree, population health goals [42]. Moreover, there is little evidence of substantial central pressure for their involvement in long-term prevention initiatives [43]. Evaluations suggest that leadership development programmes undertaken by NHS managers and clinicians tend to focus on systems leadership and address change management in service delivery, quality or training rather than in embracing new approaches to integrating prevention into NHS services [44]. To have a significant impact on population health, NHS leaders will need to embrace a concept of systems leadership that goes beyond the health care system and acknowledge the importance of the wider and more complex social determinants of health and thus the need to work with multiple stakeholders (e.g. local authorities, education providers, criminal justice system, private sector) across their local systems [45].

“Promoting health through the organised efforts of society” is fundamental to the role of public health [46]. The shift of the local public health function from the NHS back to local authorities in England in 2013 is generally agreed to be good in principle, as local authorities are well placed to address the wider social determinants of health. However, it has proved a barrier to the NHS embracing prevention as the NHS lost the direct employment of most of its public health experts. It has also naturally created a challenge for engaging the NHS in public health objectives, for which there is opportunity to address this. The lack of a public health perspective in the NHS has been recognised and there now exists Regional Directors of Public Health [47].

#### The advent of integrated care systems

**Fig. 1:**
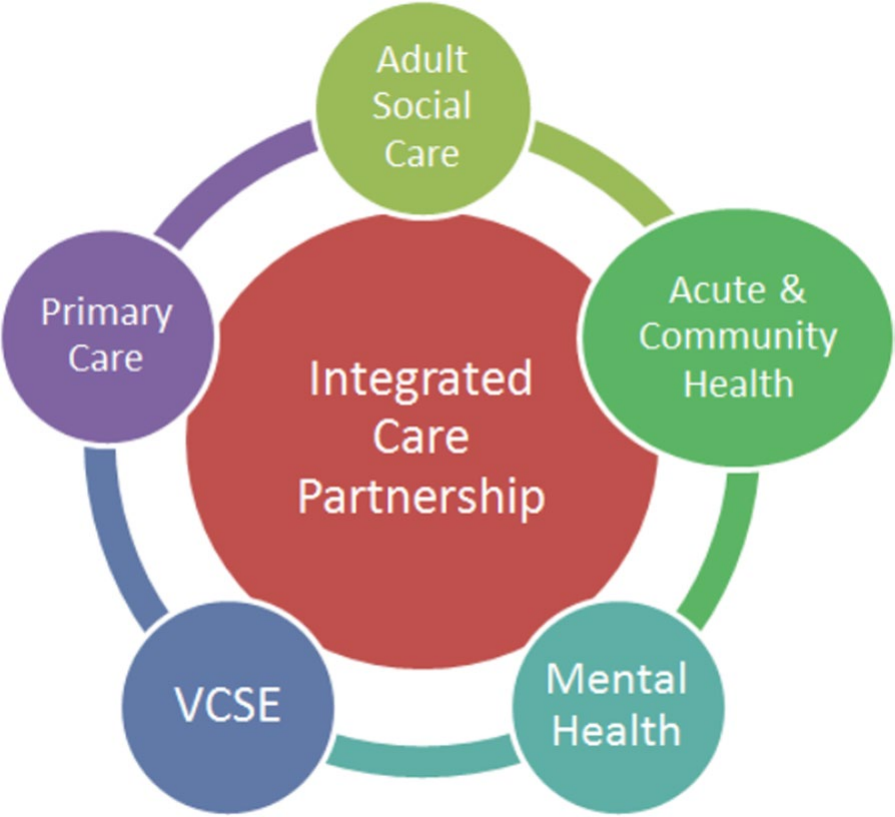
The Integrated Care System - A Model [Source: 49]

The *NHS Long Term Plan* [36] strongly expressed the policy intention to shift the focus of local NHS organisations to population health management through the establishment of integrated care systems (ICSs) covering the whole of England [48]. ICSs are intended to integrate across primary and specialist care, physical and mental health, and health and social care. In the absence of an agreed national model of the ICS, Fig. 1 presents a model of the integrated care system in Bolton, England [49]. In addition, there is a strong emphasis on prevention, although much of the focus is on secondary prevention for conditions including diabetes, obesity and respiratory illness. There is some attention given to the need for upstream interventions such as tackling air pollution, but little indication of a whole systems approach to primary prevention and no mention of the need to promote physical activity.

Similarly, the White Paper *Integration and Innovation* [50] states a clear commitment to integration of prevention and health and care services. However, as above, the prevention agenda is more focused on secondary rather than primary prevention. In particular, action on obesity is given a high profile, but there is no reference to physical activity nor is the action proposed on obesity reflective of a whole systems approach. By contrast, the Advancing Our Health: prevention in the 2020s Paper [16] emphasises the potential for ICSs to contribute to both primary and secondary prevention.

It is clear that ICSs are intended to take a whole systems approach to prevention and the integration of health and care services [51]. This is reaffirmed in the government’s policy paper *Transforming the public health system: reforming the public health system for the challenges of our times*, [52] which encouragingly signals the intention to strengthen the NHS role in prevention of ill-health, and articulates the requirement for ICSs to partner with non-NHS bodies when it comes to population health and meeting local needs. This policy paper also helps to set out the future for health improvement and public health and, at a national level, sets the scene for the recent establishment of the Office for Health Improvement and Disparities.

#### The project to engage NHS systems leaders in whole systems approaches to physical activity

Despite health care being one of the top eight best investments for increasing physical activity, [53] there are few published studies that examine how to involve health system leaders in whole systems approaches, [54] and none we identified which address how to involve the NHS in promoting physical activity in particular. To help address this lack of evidence, Public Health England commissioned the University of the West of England, Bristol (UWE Bristol) to carry out qualitative research exploring experiences of engaging NHS leaders in whole systems approaches to physical activity. This project has been reported elsewhere, [54] but briefly, eight interviews were conducted with national stakeholders working across England in different parts of the system, including for example, those from NHS England and NHS Improvement (NHSE&I) and the Local Government Association. The aim of these interviews was to gain a high-level overview of NHS engagement in whole systems activity. National interviews were complemented by interviews with 22 local informants from four case study sites in England that were identified as implementing whole systems approaches to physical activity. Two case study sites were Local Delivery Pilots (LDPs) and two were not. The four local practice examples presented diverse contexts and approaches to engaging NHS leaders in whole systems approaches to physical activity, but all had done so to different degrees. Local informants from each case study site were purposively sampled to represent different levels of the local system. Data were thematically analysed [55] and ten key themes identified (Additional File 1). The study was granted ethical approval by the University of the West of England Research Ethics Committee (Ref: HAS.20.01.095).

## Main text

### Opportunities for action in England

In our original study [54] we identified a number of common enablers and common barriers to engaging NHS systems leaders in whole systems approaches to physical activity. These are summarised in Table 1. For the purposes of this commentary, we build upon the barriers and enablers presented in Table 1 and identify opportunities for action to engage health system leaders in whole systems approaches to physical activity in England, at least in the short to medium term.

**Table 1:** Common enablers and barriers to engaging NHS leaders in whole systems approaches to physical activity (Adapted from: Evans et al., 2020) [52]

**Enablers**	**Description**
Recognising and facilitating shared system leadership	An explicit commitment to both a whole systems approach to physical and to facilitating shared systems leadership.
Key individuals or core teams championing the whole systems approach	Although overall leadership needs to be shared, motivated individuals or small core teams working across local authorities and the NHS (particularly in CCGs) can make a difference in championing a whole systems approach to physical activity.
Key facilitation role for public health across the system	All actors bring different knowledge and skills to the table, but public health professionals uniquely have the evidence-based knowledge about effective physical activity interventions, the understanding of systems approaches, the partnership skills and the remit to play a key facilitation role in promoting physical activity across sectors including in the NHS.
Balancing senior buy-in with working with enthusiasts	Gaining ‘buy-in’ from senior managers in the NHS is important (particularly to secure any necessary financial support) but it is recognised that senior managers often or usually have little time to personally engage, and once they have given broad approval for physical activity work, they might have relatively little engagement with or knowledge of the detail. Achieving real change often a result of working opportunistically with enthusiasts, whatever their formal status or level of authority whilst securing in principle support and buy-in from those in more senior leadership positions.
Taking time to build relationships and develop a shared vision	The importance of relationship building (including the time it takes build trust in relationships) and developing shared vision and shared values.
**Barriers**	**Description**
Capacity – for the NHS and other sectors	Members of the NHS system – and other sectors – have many competing priorities, and their capacity to engage with the physical activity agenda is often experienced as limited.
NHS culture	NHS performance management is traditionally focused on clinical treatment and care, quality and finance, and despite the stated commitment to prevention does not currently encourage a focus on the long-term benefits of promoting physical activity
Engaging the acute sector	Promoting physical activity is still something that is not usually seen as acute sector ‘core’ business.
Difficulty in demonstrating quantifiable impact in the short to medium term	The main benefits of greater physical activity are likely to accrue over a long-time period – 5 to 10 years or more – whilst the NHS is looking for short term reductions in indicators like bed days.
Difficulty in seeing opportunities for innovation that do not involve significant new resources	A lack of resources is not necessarily a barrier to innovation around physical activity, but there is a perception that stakeholders in the NHS and other parts of the system perceive this as an important barrier.

*CCG* Clinical Commissioning Group, *GP* General Practice, *NHS* National Health Service

The advent of ICSs in England presents an opportunity to begin to address three barriers identified in our research, namely: *Capacity of the NHS and other sectors*; *NHS culture*; and, *Engaging the acute sector* (Table 1). Whilst there is positive local practice relating to physical activity and systems approaches, to build upon as demonstrated through for example the LDPs, there remains ample opportunity to spread this learning more broadly to NHS systems and their leaders. Engaging NHS leaders will require collaborative approaches at national, regional and local levels, including between organisations such OHID and Sport England. Consideration is also required as to how to place more emphasis on promoting physical activity to support population health. A recent scoping review noted the importance of how physical activity messages are communicated, finding consistent support for the role of positive framing of physical activity messages, highlighting the short-term outcomes of physical activity, and tailoring/targeting of message content [56].

Recognition of the need to address inequalities in physical activity for people with health conditions, and support them to be active for good health and wellbeing, and to help manage their conditions has increased across health and physical activity sectors. The Richmond Group of Charities working across health and social care has established a ‘Movement for all’ programme and the ‘We Are Undefeatable’ campaign to promote physical activity to people with long term conditions. A recent consensus statement on physical activity risk endorsed by these charities recommended that the benefits outweigh the risks for most people with health conditions to get active [57]. Sport England has also identified people with long-term conditions as a priority group in their latest action plan [58]. Investment in social prescribing services – signposting people to non-clinical community based interventions – provides an opportunity, with regional Physical Activity Advisors appointed to support and co-ordinate [59].

Placing stronger emphasis on how physical activity is promoted could help to engage NHS systems leaders with specific physical activity initiatives and wider systemic prevention partnerships; once NHS systems leaders sign up to such engagement, their directors and other senior staff are likely to follow. It may also provide an opportunity for innovation that does not involve significant new resources – a barrier identified in our research (Table 1). Possible strategies could involve information sharing at key NHS webinars, events and conferences and in NHS policies and guidance, and/or the development and dissemination of tailored easily accessible resources on physical activity that would support conversations with different audiences within the NHS.

There is a need to be flexible in accordance with the structures, priorities and roles of systems at national, regional and local levels. As previously highlighted in the Global Activity Plan on Physical Activity [7] our research also identified that partnerships are key and are likely to be influenced and supported by a range of stakeholders and organisations, therefore whilst one or multiple organisations may take a lead there will be a need for shared ownership and action across organisations and level. If such collaboration can be achieved it could build upon two key enablers identified in our research (Table 1): *Recognising and facilitating shared system leadership* and *Taking time to build relationships and develop a shared vision*.

National actors (for example, Sport England, NHS England, health professional bodies, Active Partnership Network, the Local Government Association and OHID) need to collaborate, harness the evidence and practice on the benefits of systems approaches to support and influence change at the national policy level. Recent research, however, has suggested that the good intentions expressed in policy are not always carried through into implementation and they tend to have an individualistic rather than a whole systems perspective [60, 61]; the opposite approach to those expressed in key policy drivers and strategies advocating for physical activity [7, 8]. Regional and local actors like ICSs, local authorities and Active Partnerships are therefore a pivotal part of a broad-brush alliance to influence government policy towards a more systems approach to physical activity in the NHS. A critical point for consideration is how to continue co-working with the recently established OHID and take forward its commitment to support whole systems working across all its prevention agendas, including physical activity. It is vital that efforts are continued at a national and local level, when it comes to exploring and enabling delivery on whole systems working on physical activity.

The most recent White Paper [50] and the changes that will follow presents another fruitful opportunity to begin to address the barriers discussed above (Table 1: *Capacity of the NHS and other sectors*; *NHS culture*; and, *Engaging the acute sector*). Whenever there is major system change, as there will be following the White Paper, there is an opportunity to influence the direction of change. Moreover, given the impact of COVID-19 on the NHS system, the learning gleaned from systems approaches and the recovery to follow, again creates opportunities as the system realigns to engage prevention as part of the solution. The White Paper explicitly calls for greater innovation, flexibility, collaborative working and population health focus – all elements conducive to a whole systems approach to physical activity. The challenge will be in giving the whole systems approach a strong voice amongst all the other competing demands and priorities ICSs will be grappling with. Physical activity stakeholders will need to engage at all levels of the system – OHID and the newly merged NHS England (for example, embedding strong whole systems approaches to prevention in guidance to ICSs) will need to influence the ICSs directly.

A key strategic action to consider is how to rebuild and enhance public health integration in and with the NHS so that there are increasing opportunities to support *Key individuals or core team championing of whole systems approaches for physical activity* (Table 1). Our research highlighted the important impact that individual champions of physical activity can make in influencing NHS leaders at every level towards whole systems working. Systems leadership is, by definition, about collaborative and distributed leadership, but individuals can still make a difference as catalysts for action within complex systems. Scaling up and extending across England, the pockets of positive local practice, where individuals and public health teams work cohesively with the NHS, is therefore crucial for addressing the wider determinants of health and for promoting everyday physical activity. Successfully doing so will, however, mean that it is important to address barriers including *Capacity of the NHS and other sectors* and *NHS culture* (Table 1). For example, prospective champions for physical activity in the NHS, including managers and clinicians, will need to be creative; and use any spare capacity to seek collaboration with public health teams and combine their skills and experience to champion a whole systems approach. The recent ICS guidance [48] requires the ICS to integrate a public health voice at the ‘place’ level, which facilitates the role of the Director of Public Health. Whilst these developments will help to establish a platform to drive prevention and place it at the heart of ICS thinking, there remains opportunity to strengthen the tools to support systems thinking. Such efforts would build upon an enabler identified in our research: *Key facilitation role for public health across the system* (Table 1).

Our research identified the importance of *Balancing senior buy-in with working with enthusiasts* (Table 1). In these COVID-19 times, NHS leaders will inevitably have many competing demands, so ensuring action on physical activity becomes a priority in the NHS will require the continued championing of whole systems approaches by stakeholder organisations (including Sport England, the Faculty of Public Health, the Local Government Association and national government bodies) and other enthusiasts. NHS leaders can be supported in this work by the reality that physical activity is high up the current political agenda with a focus on active transport and the need to address current levels of obesity. A key argument will be to use evidence on the unintended negative impact of COVID-19 on physical activity levels as a catalyst for change to embed and align whole systems approaches to physical activity within the structures of the ICS and NHS performance management processes. Taking such action would begin to address three barriers identified in our research: *Difficulty in demonstrating quantifiable impact in the short to medium term*; and, *Engaging the acute sector*; and, *NHS culture*.

To further build upon the enablers identified above, stakeholder organisations can support regional and local partnerships to adopt whole systems approaches to physical activity, as demonstrated through the journeys of the twelve local delivery pilots (LDPs) [30]. Within the NHS, ICSs are the obvious vehicle to embed a whole systems approach to physical activity and there is much learning from the LDPs and others’ experiences to enable the health community to make this happen. All ICSs will have a population health management programme that should embed a whole systems approach to prevention, working closely with public health in local authorities, and recognise the important role of physical activity as a key part of a holistic and joined-up approach in commissioning plans. Where they exist, Active Partnerships (https://www.activepartnerships.org/) can also play a key role in facilitating a whole systems approach at the health community level.

At a neighbourhood level in health and wellbeing boards, NHS trusts and primary care networks, there will be a critical role for local authority public health departments. Local authority public health specialists will need to continue to provide the champions and facilitators for whole systems working on prevention in general and physical activity in particular at the ICS level. The COVID-19 pandemic and living with COVID-19 will remain the context within which the system is working for some time to come. Whilst this has undoubtedly challenged the public health and NHS workforce, there has been reference to the importance of systems approaches in the response to COVID-19 [62] and it would seem relevant for systems approaches to underpin recovery. This should provide opportunities for the NHS to further its leadership in health improvement and areas like physical activity and thus build upon enablers *Key facilitation role for public health across the system* and *Key individuals or core teams championing the whole systems approach* identified in our research (Table 1).

At every organisational level in England (national, regional, local), there is a need to build the evidence base around what works in whole systems working on physical activity. OHID and academic institutions among others have begun to develop this evidence [4, 23] but there is much more to do. Academic researchers can play a vital role in further evaluating whole systems approaches to physical activity and disseminating the results, in particular to demonstrate to NHS leaders the value of such approaches to the NHS. There is also the opportunity to learn from the wider literature on systems working and systems leadership in public health and synthesise this learning through systematic reviews and apply to physical activity work. Building a shared approach and understanding between academic researchers and policy makers and NHS leaders will not be without its challenges. However, different actors have what can appear to be conflicting needs; NHS leaders and policy makers operate in an often pressurised environment where they need answers immediately whilst academic research often requires years to come to robust conclusions [24]. As Rutter and colleagues make clear, there is no simple mechanism to address this challenge, but seeing it as part of the whole system approach is a start.

Changing the culture of the NHS to embrace whole systems working also has educational implications for the NHS Leadership Academy, the Medical Royal Colleges, Health Education England, universities, and others concerned with the education of NHS leaders, managers and senior clinicians. One example of good practice is a collaborative project that is already underway between the Royal College of General Practitioners and Sport England [63]. The Active Practice Charter seeks to inspire and celebrate practices that are taking steps to increase activity and reduce sedentary behaviour in their patients and staff. This offers a real opportunity to embed physical activity into care pathways in a way that could support a whole systems approach and to directly address *NHS culture* which does not currently encourage a focus on physical activity; a key barrier identified in our research (Table 1).

## Conclusions

Promoting physical activity and contributing to whole systems approaches to physical activity is crucial for health systems improving population health and managing demand, including for the NHS in England. There are pockets of good practice from which lessons can be learned, but there is a need for whole systems approaches as part of a broader, holistic, prevention agenda. Action is required at all levels, particularly in identifying and addressing underlying system barriers, to make participating in whole systems action in the NHS a reality. Engagement of NHS leaders with physical activity at a system level remains variable and so it is vital to consider, within national structures and at a local level, how this can be changed, and which drivers are needed. The White Paper on NHS integration and innovation, in addition to development of Integrated Care Systems and Sport England’s *Uniting the Movement* strategy, provide a platform to address this. Furthermore, actors including Sport England, NHS England, health professional bodies, Active Partnerships, the Local Government Association and OHID, need to collaborate, harness the evidence and practice on the benefits of systems approaches to support and influence change. These organisations also need to continue to enable and support local physical activity partnerships to engage the NHS and local authorities. Finally, public health specialists have a key role as champions and facilitators of whole systems approaches to prevention in general and physical activity in particular.

## Supplementary Information

**Additional file 1.**

## Data Availability

This study does not have ethical approval to make the study dataset publicly available. Data may be available from the corresponding author on reasonable request.

## Abbreviations

GAPPA: Global Action Plan on Physical Activity
ICS: Integrated care system
LDPs: Local delivery pilot
NHS: National Health Service
OHID: Office for Health Improvement and Disparities
WHO: World Health Organization
PHE: Public Health England
