# Supplementary material for: Opportunities to engage health system leaders in whole systems approaches to physical activity in England
Source: BMC Public Health. 2022 Feb 8;22:254. doi: 10.1186/s12889-022-12602-5 (PMC8822681; doi:10.1186/s12889-022-12602-5)
Supplement: Supplementary file 1 — Additional file 1. [file 12889_2022_12602_MOESM1_ESM.docx]

Additional File 1. Engaging NHS leaders in whole systems approaches to physical activity: key themes

| The importance of shared vision |
| --- |
| Collective systems leadership is crucial, not individual system leaders |
| Systems leadership is needed at all levels of the system |
| Place-level is a crucial focus for physical activity work |
| Relationships are crucial; relationship building really important |
| Enthusiasm and commitment are as important as formal seniority |
| There is a key facilitation role on physical activity for public health across the system |
| A range of resources is required |
| Central pressures are driving siloed working |
| Quantifiable outcomes are wanted in unrealistic short timescales |
